# Fracture Properties and Softening Curves of Steel Fiber-Reinforced Slag-Based Geopolymer Mortar and Concrete

**DOI:** 10.3390/ma11081445

**Published:** 2018-08-15

**Authors:** Yao Ding, Yu-Lei Bai

**Affiliations:** 1 College of Civil Engineering, Chongqing University, Chongqing 400044, China; dingyaohit@126.com; 2 Key Laboratory of Urban Security and Disaster Engineering of Ministry of Education, Beijing University of Technology, Beijing 100124, China

**Keywords:** slag-based geopolymer, concrete, mortar, short steel fibers, fracture property, softening curve

## Abstract

Adding short steel fibers into slag-based geopolymer mortar and concrete is an effective method to enhance their mechanical properties. The fracture properties of steel fiber-reinforced slag-based geopolymer concrete/mortar (SGC/SGM) and unreinforced control samples were compared through three-point bending (TPB) tests. The influences of steel fiber volume contents (1.0%, 1.5% and 2.0%) on the fracture properties of SGC and SGM were studied. Load-midspan deflection (*P-δ*) curves and load-crack mouth opening displacement (*P*-CMOD) curves of the tested beams were recorded. The compressive and splitting tensile strengths were also tested. The fracture energy, flexural strength parameters, and fracture toughness of steel fiber-reinforced SGC and SGM were calculated and analyzed. The softening curves of steel fiber-reinforced SGC and SGM were determined using inverse analysis. The experimental results show that the splitting tensile strength, fracture energy, and fracture toughness are significantly enhanced with fiber incorporation. A strong correlation between the equivalent and residual flexural strengths is also observed. In addition, the trilinear strain-softening curves obtained by inverse analysis predict well of the load-displacement curves recorded from TPB tests.

## 1. Introduction

Although ordinary Portland cement concrete (PCC) is regarded as the most widely used construction material, and some novel cementitious composites featuring outstanding properties have been proposed recently [1,2,3], their inherent adverse effects on the environment are attracting increasing attention. The emphasis on sustainable development has motivated researchers to explore new cementitious materials as partial or complete alternatives to Portland cement (PC) [4,5,6,7]. Geopolymer cement has recently been regarded as a potential alternative to PC.

Slag-based geopolymer utilizes ground granulated blast furnace slag (GGBFS) as the sole raw material, and is activated by alkali solutions to form cementitious material. It was first studied by Purdon [8], and extensive studies have subsequently demonstrated that slag-based geopolymer exhibits similar mechanical properties to or even performs better than PC in many aspects [9,10].

Despite its many advantages, such as early strength development, durability, high resistance to chemical attack, low hydration heat and good resistance to freeze-thaw cycles [9,10], slag-based geopolymer still exhibits a brittle nature, similar to that of PC. In addition, its autogenous and drying shrinkages are 4–5 times larger than those of PC [11,12,13,14,15], which would further decrease its ability to resist fracture. Several studies have demonstrated that the incorporation of fibers enables cracking control and efficiently improves its mechanical properties, including the tensile and flexural strength of slag-based geopolymer [16,17,18], through the fiber bridging effect. In addition, fiber inclusion can obviously alleviate the shrinkage of slag-based geopolymer [18,19,20]. It is known that the fracture properties of fiber-reinforced concrete are generally governed by the size and angularity of the coarse aggregates, the microstructure of the paste, the interfacial transition zones (ITZs) between the aggregates and the paste [21,22,23] as well as between the fiber and the paste [24], and the properties of the fibers. Previous research has stated that the ITZs in slag-based geopolymer concrete (SGC) are denser and less porous than those in PCC [25,26], and the bonding performances of steel fiber with these two series of matrices are not the same [17,27]. Therefore, the fracture properties of fiber-reinforced SGC and PCC are believed to be different. Although many studies have been conducted on fiber-reinforced PCC [28,29], limited studies have been conducted to study the influence of steel fibers on the fracture properties of slag-based geopolymer. Bernal et al. [17] found that the fracture toughness of SGC increased with the steel fiber dosage and the reinforcement of steel fiber in SGC was more efficient than that in PCC. Aydın and Baradan [18] stated that the toughness of slag/silica fume-based geopolymer mortar with steel fiber incorporation increased up to 125 times compared to control mortar without fiber. Similarly, slag/silica fume-based geopolymer mortar presented significantly better mechanical performance than PC mortar with the same fiber dosage. Bhutta et al. [30] found that hooked-end steel fiber-reinforced fly ash-based geopolymer mortar with a fiber volume addition of 0.5% exhibited the most ductile flexural behavior compared to other steel fibers (length deformed and straight) in both heat and ambient curing. However, due to the insufficiency of the existing experimental data, post-cracking behavior, including the tension softening behavior of steel fiber-reinforced SGM and SGC, has rarely been studied.

The aim of this paper is to further study the effect of steel fiber volume contents on the mechanical and fracture properties of SGM and SGC. Three fiber volume fractions, including 1.0%, 1.5% and 2.0%, were utilized to reinforce both SGM and SGC. Unreinforced SGM and SGC specimens were tested as control samples. The compressive and splitting tensile strengths were also tested. Three-point bending (TPB) tests were conducted following the RILEM TC50-FMC [31] recommendation. The tension softening curves of steel fiber-reinforced SGM and SGC were determined using inverse analysis. Then, the load-displacement curves of steel fiber-reinforced SGM and SGC obtained directly from TPB tests and those predicted by using the softening curves were compared. In addition, the fracture energy, equivalent and residual flexural strengths, and fracture toughness were calculated and compared.

## 2. Experiment Program

### 2.1. Constituent Materials

The ground granulated blast furnace slag (GGBFS) used in this study was from Nanjing, China, and its chemical composition measured by X-ray fluorescence (XRF, ARL, ADVANT) analysis, as shown in Table 1. The particle size distribution of GGBFS mainly ranged from 0.4 μm to 100 μm, and the morphology of GGBFS particles was predominately of anomalous shape with clear edges and angles.

The alkali activator liquid used was a mixture of sodium silicate solution and sodium hydroxide. The water content and the modulus (the mole ratio of SiO_2_ to Na_2_O) of the sodium silicate solution were 59% (by mass) and 3.7, respectively. The addition of 99% pure sodium hydroxide (NaOH) flakes helped to adjust the modulus of the alkali activator to the targeted values.

**Figure 1:**
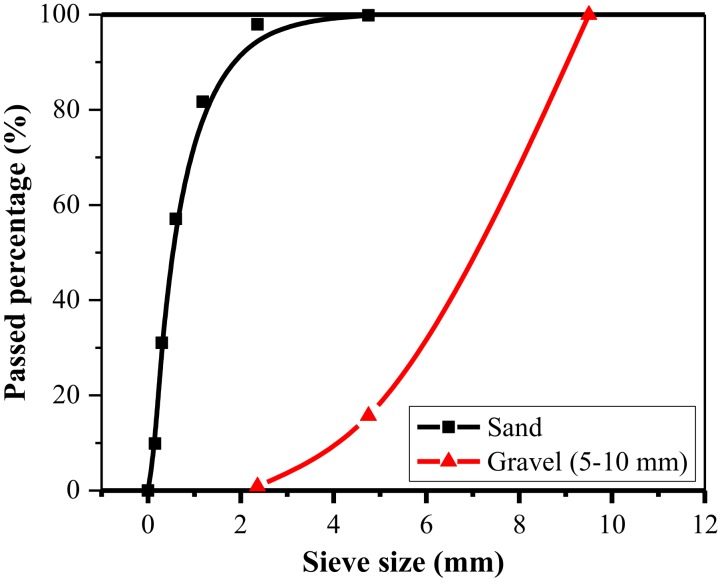
Gradation curves of fine and coarse aggregates.

The fine aggregate was medium sand with a fineness modulus of 2.81. Additionally, gravel from the local river with a maximum size of 10 mm was selected as the coarse aggregate. The bulk specific density and water absorption of the coarse aggregate were 2530 kg/m^3^ and 1.83%, respectively. The grading curves of the coarse and fine aggregates are given in Figure 1.

The basic properties and configuration of the hooked-end deformed steel fiber used in this study are listed in Table 2. The length of the steel fiber was 13 mm and its aspect ratio was 60.

### 2.2. Mix Proportion

The mix proportions of unreinforced SGM and SGC are summarized in Table 3. The alkali concentration (the percentage of Na_2_O by mass of slag, *n*) and the modulus of the alkali activator (the mole ratio of SiO_2_ to Na_2_O, *M_s_*) were determined based on former research conducted by the authors [32,33] in order to guarantee the workability of the slag-based geopolymer matrix [9,34]. The steel fiber volume contents were 1.0%, 1.5% and 2.0% for both SGM and SGC. The fiber-reinforced SGM and SGC mixes were designated based on their fiber content (shown in Table 4). For example, SGM-SF1.0 represents SGM with steel fiber reinforcement of 1.0%.

### 2.3. Sample Preparation

The sodium hydroxide, sodium silicate solution and water were firstly blended to form an alkali activator solution 24 h before concrete mixing to ensure that the solution cooled down to room temperature. The weighted GGBFS, fine aggregate and coarse aggregate were added into the mixer and dry-mixed for 3 min. Then, the alkali activator solution was slowly poured into the mixer and mixed with the solid fraction for another 3 min. Once a consistent mixture was reached, the fibers were added slowly and mixing was continued until uniform dispersion could be observed. The final mixture was cast into the prepared molds and solidified on a vibrating table. All the specimens were covered with plastic sheets for 24 h and then demolded and cured in an environmental chamber with a constant temperature of 21 ± 1 °C and a relative humidity of 90 ± 5% for 28 days.

### 2.4. Testing Procedure

#### 2.4.1. Compressive and Splitting Tensile Strength Tests

Compressive strength and splitting tensile strength were tested using a universal testing machine of 2000 kN capacity. The specimens used were 70.7 mm cubes and 150 mm cubes for mortar and concrete, respectively, for testing both the compressive and splitting tensile strengths. Three and six identical specimens were prepared for compressive and splitting tensile strength tests, respectively, for each type of mixture. The loading rate adopted in the compressive strength test was 0.8 MPa/s, while the loading rate for the splitting tensile strength test was 0.08 MPa/s [35].

#### 2.4.2. Three-Point Bending (TPB) Test

**Figure 2:**
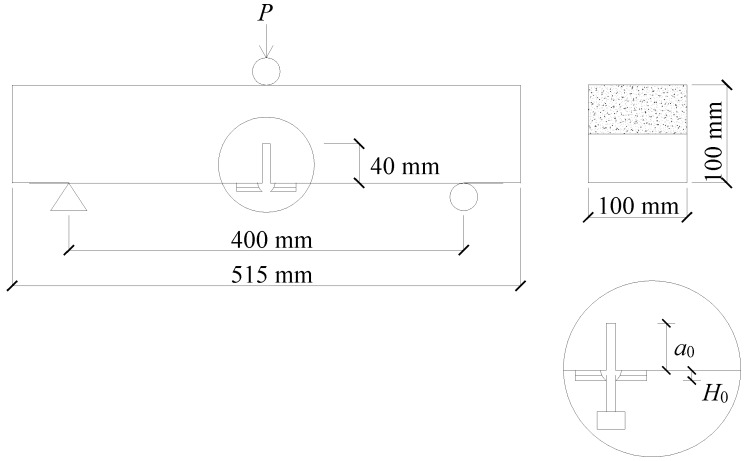
Configuration of TPB test beams.

The beam sizes used for the three-point bending (TPB) tests were 100 mm × 100 mm × 515 mm. The span-to-depth ratio was 4.0. All the specimens were precut in the middle of the beams with a notch of 40 mm height and 3 mm width. The beam was simply supported with the notched face down. The geometry of the specimen is shown in Figure 2. Four identical specimens were prepared for each mixture. The crack mouth opening displacement (CMOD) was measured using the clip gauge clamped at the mouth of the precut notch. Two linear variable differential transformers (LVDTs) were used to detect the midspan displacement (*δ*) of the beam, and the effect of support settlement on the midspan displacement was removed by measuring the displacements of the two supports simultaneously. A closed-loop servo-controlled hydraulic jack with 100 kN capacity was used. The loading rate for the unreinforced specimens was kept at 0.02 mm/min [36], while it was settled at 0.5 mm/min for the reinforced ones [37]. TPB tests on the notched beams were conducted to determine the fracture energy [31], equivalent and residual flexural strengths [37], and fracture toughness of the specimens [38].

## 3. Testing Results and Discussion

### 3.1. Experimental Phenomena

**Figure 3:**
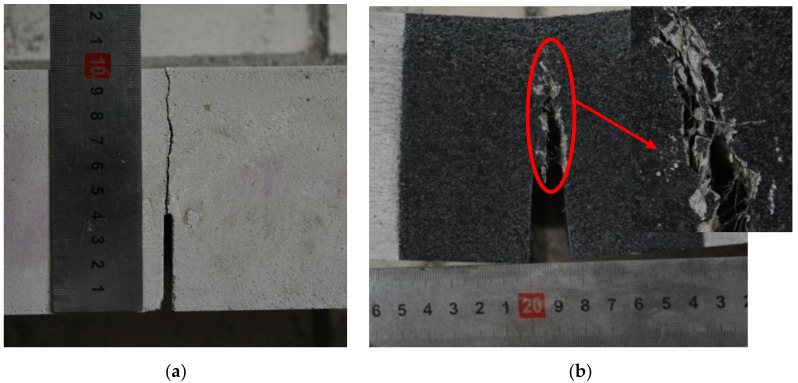
Failure modes of plain and steel fiber-reinforced SGM. (**a**) Plain SGM; (**b**) Steel fiber-reinforced SGM.

For plain SGM and SGC beams, once the crack initiated, the maximum load was reached in a very short time; no visible cracks could be observed at this stage. The beam lost the capacity to withstand more load soon after the peak load was achieved. The crack propagated in a straight line from the notch tip to the top of the beam (Figure 3a) and the fracture surface was smooth. These phenomena demonstrated the brittleness of plain SGM and SGC.

The ductile failure mode was observed for steel fiber-reinforced SGM and SGC beams. When the fibers were added into the matrix, the beam could keep deforming and withstanding load even after the peak load was reached. The mid-span deformations of the beams at failure were more than 10 mm, which were significantly larger than those of the plain specimens. Numerous tiny cracks could be observed near the main crack due to the fiber bridging effect (Figure 3b). Hence, the ductility of the fiber-reinforced specimens was much better than that of the plain ones.

### 3.2. Compressive and Splitting Tensile Strengths

The average 28-day compressive strengths of plain and steel fiber-reinforced SGM and SGC are listed in Table 4. It can be seen that the average compressive strength (*f_c_*) of plain SGM was 69.2 MPa, while the enhancement of the compressive strength was 13%, 13% and 22%, respectively, with the fiber volume additions of 1.0%, 1.5% and 2.0%, which was similar to previous observations [18]. The strength enhancement can be attributed to the ability of the fibers to transfer stresses and loads [39,40]. With regard to SGC, the reinforcement effect with fiber incorporation reached its maximum (i.e., a 22% increase) when the fiber volume fraction was 1.5%. However, further increase of the fiber dosage to 2% did not continuously improve the compressive strength of SGC. This result was consistent with previous research conducted on steel fiber-reinforced high-strength concrete [41]. This might be due to the fact that the overdose of steel fiber would cause mixing difficulty and fiber balling, which would generate adverse influences on workability and uniformity. Therefore, a less obvious improvement in the compressive strength was observed when fiber content was increased to 2%.

Compared with the enhancement to the compressive strength of SGM and SGC with steel fiber incorporation, the reinforcement efficiency on the splitting tensile strength (*f_st_*) was more significant, as displayed in Table 4, which is consistent with prior research [16,17,18]. The enhancement of the splitting tensile strength of SGM with steel fiber incorporation increased with the fiber volume fraction. The splitting tensile strength of plain SGM was 5.03 MPa, and such a value was improved to 13.14 MPa (i.e., a 161% increase) when the fiber volume addition was 2.0%. The splitting tensile strength enhancement with steel fiber addition on SGC was not as significant as that on SGM, while still showing a 23–38% increase. The reinforcement efficiency of SGC reached its best when the steel fiber volume fraction was 1.5%, which was similar to that of the compressive strength. No further strength improvement was observed when the fiber volume fraction was increased from 1.5% to 2.0%, which was largely due to the poor workability of the fiber composite, resulting in mixing difficulty and decrease in uniformity.

Xu and Shi [42] proposed an empirical relationship between the splitting tensile strength *f_st_* and the compressive strength *f_c_* of hooked-end steel fiber-reinforced cement concrete with a fiber aspect ratio of 50–80 (the fiber aspect ratio used in this study was 60) based on the collected experimental data though regression analysis, as follows:

(1) $$f_{st}=0.21f_{c}^{0.83}\;$$

**Figure 4:**
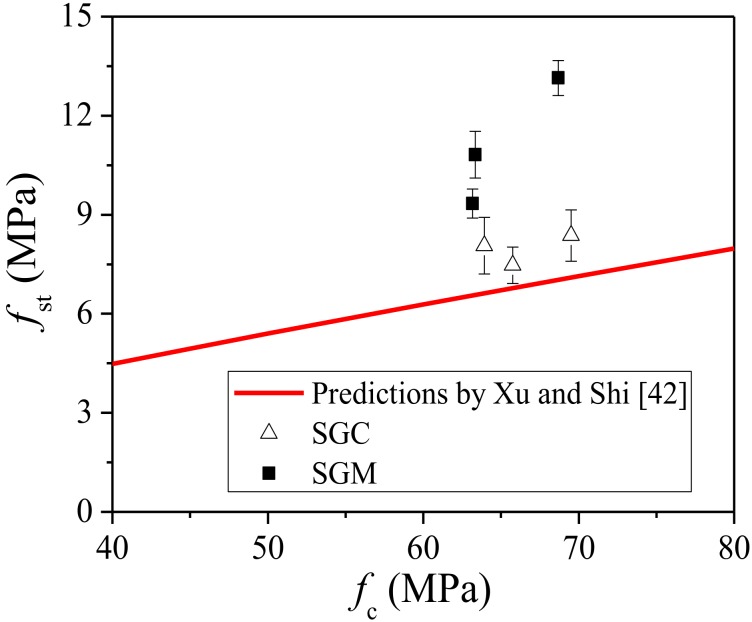
Relationship between splitting tensile strength and compressive strength of steel fiber-reinforced SGM/SGC.

The experimental results of the steel fiber-reinforced SGM and SGC obtained in this study and the predictions proposed by Xu and Shi [42] are compared in Figure 4. It is seen that the predictions underestimate the splitting tensile strength of both fiber-reinforced SGM and SGC, especially for the case of SGM. The splitting tensile strength of SGC with the fiber volume addition of 1.0% was 7.47 MPa, which was 10.3% higher than that predicted by Xu and Shi [42] (i.e., 6.77 MPa). This might be attributed to the better bond characteristics of geopolymer binders with steel reinforcement compared with PC [18,43].

### 3.3. Load-Deflection Curves and Ultimate Load

**Figure 5:**
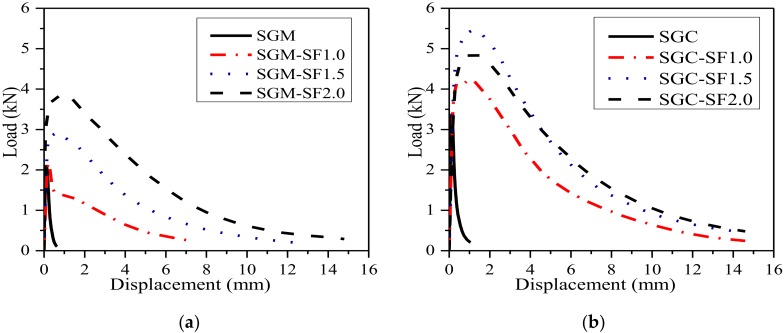
*P-δ* curves of plain and steel fiber-reinforced SGM/SGC. (**a**) SGM; (**b**) SGC.

The average *P-δ* curves of steel fiber-reinforced SGM and SGC beams along with unreinforced control specimens are shown in Figure 5a,b. The specimens without fibers failed in a sudden manner and without warning. Conversely, a non-linear elastic increase of load was found for the steel fiber-reinforced beams before the maximum load was reached. After that, the load decreased gradually with further displacement increase. The post-peak behavior was significantly improved by fiber incorporation. The incorporation of fibers clearly made contribution to enlarge the area under the load-deflection curve and improve the energy absorption capacity during the fracture process. Figure 5a shows that the mid-span deformation at the failure of steel fiber-reinforced SGM specimen with 2% fiber addition reached 14 mm, while this value was less than 1 mm for plain SGM specimen. The average *P-δ* curves of plain and steel fiber-reinforced SGC specimens with the fiber volume fractions of 1.0%, 1.5%, and 2.0% are shown in Figure 5b. It is obvious that the area enclosed by the *P-δ* curve reached its maximum when the steel fiber volume content was 1.5%, which seemed to be the optimal steel fiber volume content for SGC.

**Figure 6:**
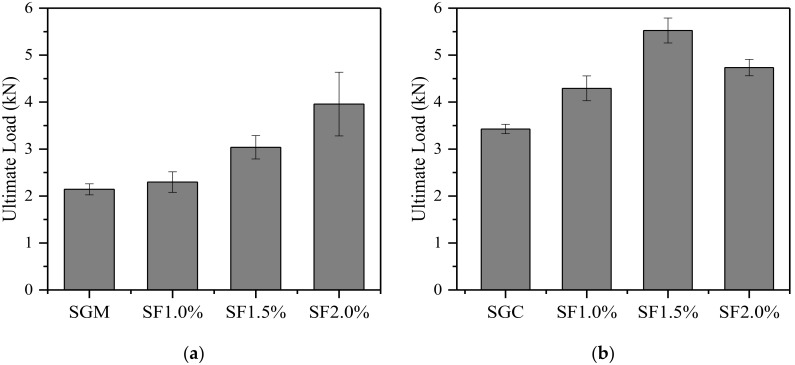
Ultimate load of plain and steel fiber-reinforced SGM/SGC. (**a**) SGM; (**b**) SGC.

Figure 6 presents the ultimate loads *P_u_* of plain and steel fiber-reinforced SGM and SGC beams obtained from the TPB tests. It is clear that the incorporation of fibers efficiently improved the ultimate loads of SGM and SGC because the fibers served as crack arrests or barriers in the matrix. The ultimate loads *P_u_* of unreinforced SGM and SGC beams were 2.1 kN and 3.4 kN, respectively. As expected, the *P_u_* of steel fiber-reinforced SGM increased with the fiber volume content (Figure 6a). The average ultimate load *P_u_* of SGM beams increased from 2.3 kN to 3.9 kN (i.e., a 69.6% increase) when the steel fiber volume content increased from 1.0% to 2.0%. As shown in Figure 6b, the enhancement of the ultimate load of SGC with steel fiber incorporation approached its largest when the fiber volume content was 1.5%. Adding more fibers did not continue improving the ultimate load of the specimens.

### 3.4. Load Fracture Properties

#### 3.4.1. Fracture Energy

The incorporation of fibers can turn concrete into a relatively high energy absorbing material, which could mitigate the hazards that structures may suffer when subjected to dynamic loads. The fracture energy of fiber-reinforced concrete specimens has to be computed with reference to a specified displacement value. A reliable cut-off point can be chosen at 10 mm [44]. The fracture energy *G_F_* can be determined indirectly based on the TPB test recommended by RILEM TC50-FMC [31] using Equation (2):

(2) $$G_{F}=\left(W_{0}+mg\delta_{0}\right)/A_{lig}\;$$

where *m* is the beam mass between two supports (kg); *W*_0_ is the external work (N∙m); *δ*_0_ is the final mid-span deformation (m); *g* is the gravitational acceleration, 9.81 m/s^2^ and *A_lig_* is the ligament area (m^2^).

**Figure 7:**
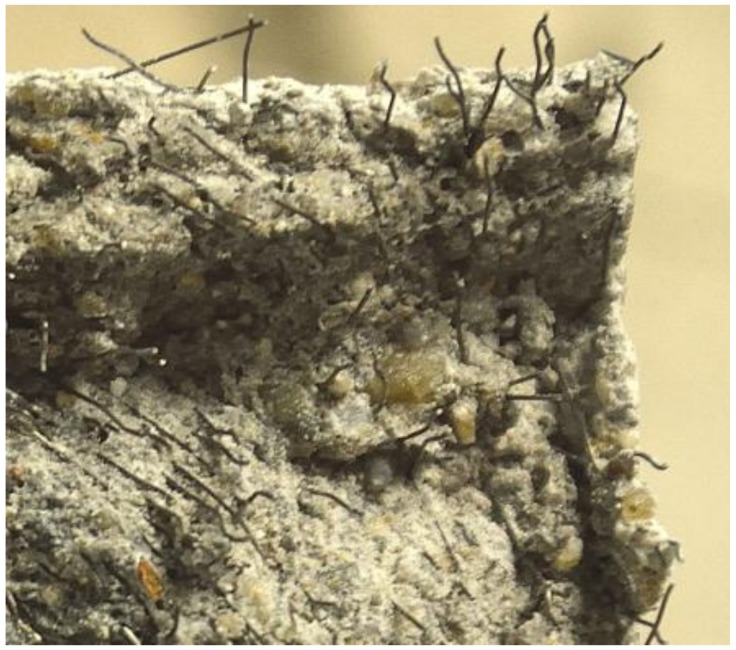
Fracture surface of steel fiber-reinforced SGM.

The average fracture energies of unreinforced and steel fiber-reinforced SGM and SGC beams are calculated and summarized in Table 5, in which *K* is an index representing the degree of improvement in the fracture energy due to fiber inclusion (i.e., *G_F_* (fiber-reinforced)/*G_F_* (non-reinforced)). It is clear that the energy absorption capacity is significantly improved with the fiber incorporation for both SGM and SGC. The average fracture energy of the unreinforced SGM beam was 97.4 N/m, while this value increased to 4188.0 N/m (i.e., about 46 times) when the steel fiber volume content was 2.0%. Observing the fracture surface of steel fiber-reinforced specimens as shown in Figure 7, it can be seen that the steel fibers were pulled out from the matrix, which is consistent with previous research [45]. The better bond characteristic of geopolymer binders with steel reinforcement [18,27,43] caused by their homogenous micro-structures [25] could result in significant fracture energy enhancement with fiber incorporation. Table 5 also illustrates that the optimum steel fiber volume fraction for SGC was 1.5%, and the fracture energy reached a maximum of 5875.2 N/m, which is about 28 times that of the control SGC beam.

#### 3.4.2. Equivalent and Residual Flexural Strengths

**Figure 8:**
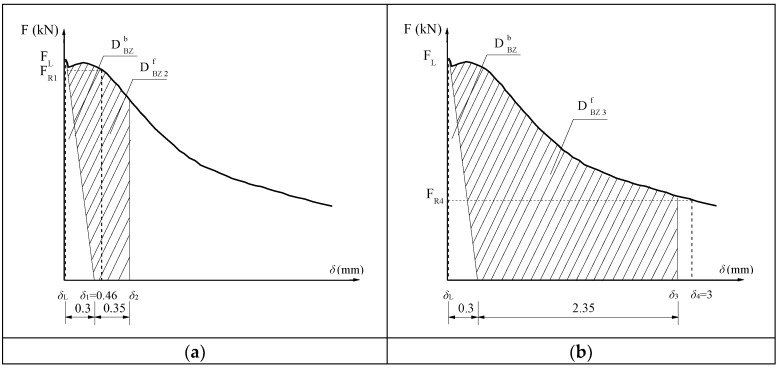
Evaluation of residual and equivalent flexural strengths (RILEM TC 162-TDF 2002). (**a**) Evaluation of *f_eq,2_* and determination of *F_R,1_*; (**b**) Evaluation of *f_eq,3_* and determination of *F_R,4_*.

According to RILEM TC 162-TDF [37], the load at the limit of proportionality *F_L_*, the corresponding strength *f_f,L_*, the equivalent flexural strength *f_eq_*_,2_ and *f_eq_*_,3_, and the residual flexural strength *f_R_*_,1_ and *f_R_*_,4_ as shown in Figure 8, were assessed with reference to the load vs. mid-span displacement curves recorded experimentally. The strength corresponding to the limit of proportionality can then be evaluated using Equation (3):

(3) $$f_{f,L}=\frac{3F_{L}S}{2B{\left(H-a_{0}\right)}^{2}}\;$$

where *B*, *H*, *S* and *a*_0_ are the thickness, depth, span and initial notch depth of the beam, respectively.

The equivalent flexural strength *f_eq_*_,2_ and *f_eq_*_,3_ were evaluated up to the deflections of *δ*_2_ and *δ*_3_ (*δ*_2_ = *δ_L_* + 0.65 and *δ*_3_ = *δ_L_* + 2.65, where *δ_L_* is the deflection corresponding to *F_L_*). The energy required by the fracture of plain concrete $D_{BZ}^{b}$ was excluded when evaluating the equivalent flexural strength. Only the effect of fibers ($D_{BZ,2}^{f}$ and $D_{BZ,3}^{f}$) was considered, as shown in Figure 8. The equivalent flexural strength can be calculated using Equations (4) and (5):

(4) $$f_{eq,2}=\frac{3S}{2B{\left(H-a_{0}\right)}^{2}}\frac{D_{BZ,2}^{f}}{0.5}\;$$

(5) $$f_{eq,3}=\frac{3S}{2B{\left(H-a_{0}\right)}^{2}}\frac{D_{BZ,3}^{f}}{2.5}\;$$

The residual flexural strength *f_R_*_,1_ and *f_R_*_,4_, referring to the midspan deflection of 0.46 mm and 3.0 mm, respectively, could be evaluated using Equations (6) and (7):

(6) $$f_{R,1}=\frac{3F_{R,1}S}{2B{\left(H-a_{0}\right)}^{2}}\;$$

(7) $$f_{R,4}=\frac{3F_{R,4}S}{2B{\left(H-a_{0}\right)}^{2}}\;$$

**Figure 9:**
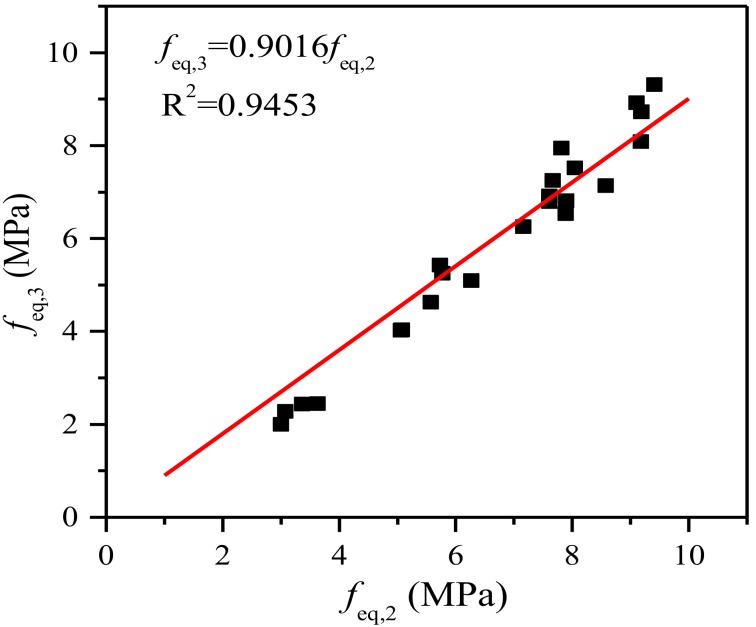
Relationship between *f_eq_*_,2_ and *f_eq_*_,3_.

**Figure 10:**
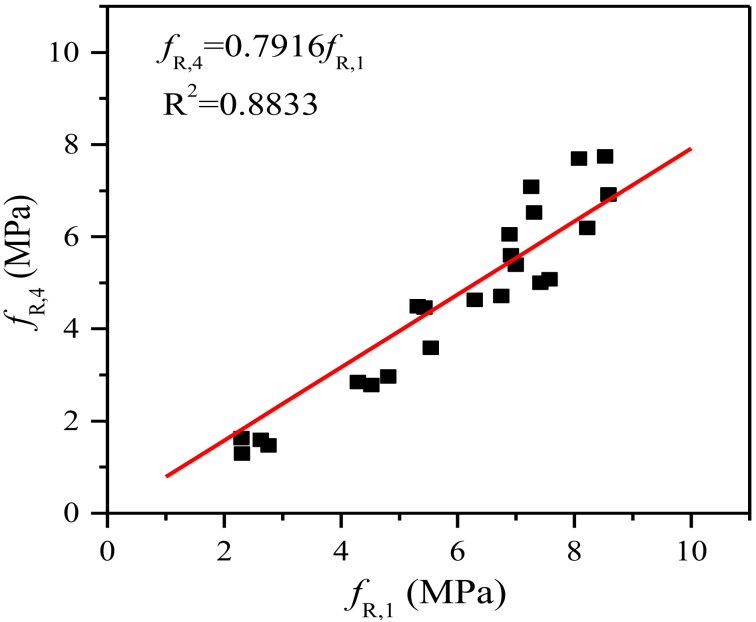
Relationship between *f_R_*_,1_ and *f_R_*_,4_.

**Figure 11:**
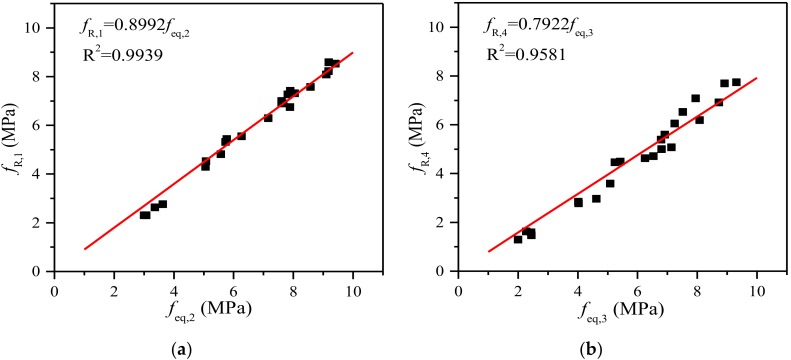
Relationship between equivalent and residual flexural strengths. (**a**) Relationship between *f_eq_*_,2_ and *f_R_*_,1_; (**b**) Relationship between *f_eq_*_,3_ and *f_R_*_,4_.

All of the above parameters of steel fiber-reinforced SGM and SGC are reported in Table 6. This reveals that the equivalent flexural strength *f_eq_*_,2_ of both SGM and SGC is higher than their other equivalent flexural strength *f_eq_*_,3_, which is consistent with the RILEM TC 162-TDF’s [37] recommendation that the former be used in serviceability state and the latter in ultimate state. The linear trend between *f_eq_*_,3_ and *f_eq_*_,2_ of steel fiber-reinforced SGM and SGC carried out by linear regression analysis is shown in Figure 9. The proportionality coefficient (i.e., 0.9016) is lower than that (i.e., 0.9926) obtained by Barros et al. [46] by conducting TPB tests on deformed steel fiber-reinforced PCC with a fiber volume addition up to 0.57%. It is clear that the residual flexural strength is easier to evaluate than the equivalent flexural strength. The relationship between *f_R_*_,1_ and *f_R_*_,4_ of steel fiber-reinforced SGM and SGC is shown in Figure 10. It is clear that *f_R_*_,4_ is about 79% of *f_R_*_,1_, which is similar to that of steel fiber-reinforced PCC [47]. In addition, the relationships between *f_eq_*_,2_ and *f_R_*_,1_ and between *f_eq_*_,3_ and *f_R_*_,4_ are presented in Figure 11. A strong correlation between the equivalent and residual flexural strength parameters is observed in fiber-reinforced SGM and SGC, which is consistent with the results obtained by other authors conducted on fiber-reinforced PCC [46,47].

#### 3.4.3. Fracture Toughness

According to the handbook of stress analysis [48], the stress intensity factor for three-point bending beams with a span-to-depth ratio of 4.0 can be determined as follows:

(8) $$K_{1}=\sigma \sqrt{\pi ag_{1}}(a/H)\;\;\sigma =\frac{3PS}{2H^{2}B}$$

(9) g1(aH)=1.99−(aH)(1−aH)(2.15−3.93(aH)+2.7(aH)2)π(1+2(aH))(1−aH)1.5 

(10) CMOD=4σaEg2(aH) σ=3PS2H2B

(11) g2(aH)=0.76−2.28(aH)+3.87(aH)2−2.04(aH)3+0.66(1−aH)2 

where *P* is the load, *CMOD* is the crack mouth opening displacement, *E* is the Young’s modulus and *a* is the crack length.

The fracture toughness of steel fiber-reinforced SGM and SGC can be calculated by Equations (8)–(11), referring to the following steps [49,50,51]:

(1) Calculate the Young’s modulus *E* using Equations (10) and (11) according to the initial cracking load *P_ini_* and the corresponding *CMOD*;
(2) Insert the measured maximum load *P*_u_ and the corresponding *CMOD*_c_, and the Young’s modulus *E* into Equation (10) to calculate the critical crack length *a_c_*;
(3) Substitute the measured maximum load *P_u_* and the evaluated *a*_c_ into Equation (8) to obtain the fracture toughness.

Dias and Thaumaturgo [49] proposed that the reinforcement effect of fibers can be represented by using the toughening factor (*F_T_*) calculated by Equation (12):

(12) $$F_{T}=\frac{K_{IC}\;\mathrm{for}\;\mathrm{fiber}-\mathrm{reinforced}\;\mathrm{concrete}}{K_{IC}\;\mathrm{for}\;\mathrm{unreinforced}\;\mathrm{concrete}}\;$$

The fracture toughness *K_IC_* and the *F_T_* values of SGM and SGC are summarized in Table 7. The fracture toughness of unreinforced SGM was 1.01 MPa·m^1/2^ and it increased with the fiber volume content. The *F_T_* of steel fiber-reinforced SGM was 4.25 when the fiber volume content was 2.0%. For steel fiber-reinforced SGC, the fracture toughness *K*_IC_ and the *F*_T_ reached the largest value when the steel fiber volume content was 1.5%.

#### 3.4.4. Softening Curves

Softening curve is a basic component of the fictitious crack model proposed by Hillerborg et al. [52]. It is a material property which represents the relationship between the cohesive stress and the corresponding crack opening across the fracture process zone (FPZ).

**Figure 12:**
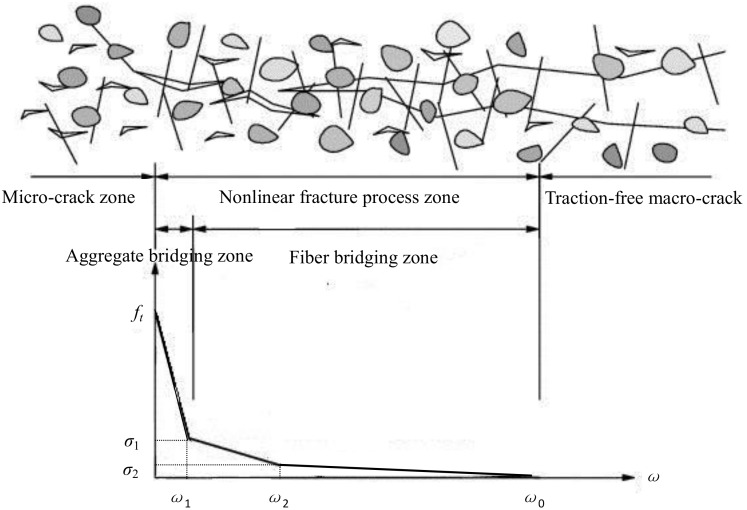
Trilinear softening traction-separation law.

In practical applications, simplified strain-softening models have been used to describe the real strain-softening diagram of concrete. Based on previous studies, several researchers [53,54,55] have suggested using a trilinear strain-softening diagram (Figure 12) to predict the load-displacement curves of fiber-reinforced concrete. The fracture mechanisms of fiber-reinforced concrete are different from those of plain concrete because of the incorporation of discrete fibers which increase the size of the FPZ [55]. The nonlinear FPZ for fiber-reinforced concrete is divided into the aggregate bridging zone and the fiber bridging zone (see in Figure 12). A general expression of the trilinear softening traction-separation law is given by Equation (13).

(13) $$\sigma (w)=\left\{ \begin{array}{l} f_{t}-(f_{t}-f_{1})\frac{w}{w_{1}}\begin{array}{ccc} & & 0\leq w\leq w_{1} \end{array}\\ f_{t}-\frac{f_{1}-f_{2}}{w_{2}-w_{1}}(w-w_{1})\begin{array}{ccc} & & w_{1}\leq w\leq w_{2} \end{array}\\ f_{2}-\frac{w-w_{2}}{w_{0}-w_{2}}f_{1}-f_{2}\begin{array}{ccc} & & w_{2}\leq w\leq w_{0} \end{array} \end{array}\right.\;$$

The above equation (Equation (13)) is characterized by six independent parameters, the tensile strength *f_t_*, two kink points (*f*_1_, *w*_1_ and *f*_2_, *w*_2_) and the crack width *w*_0_ that corresponds to zero cohesive stress.

On the basis of TPB tests, the softening law can be determined indirectly by a backward analysis [51,56,57,58]. The software CONSOFT [59,60] originally developed by Prof. Volker Slowik and his colleagues at the University of Applied Sciences in Leipzig Germany was utilized to determine the softening curves of the steel fiber-reinforced SGM and SGC.

The essential parameters of the trilinear softening curves of steel fiber-reinforced SGM and SGC obtained from the inverse analysis are summarized in Table 8. The values of *f_t_* used here were obtained from the splitting tensile tests (refer to Table 4).

**Figure 13:**
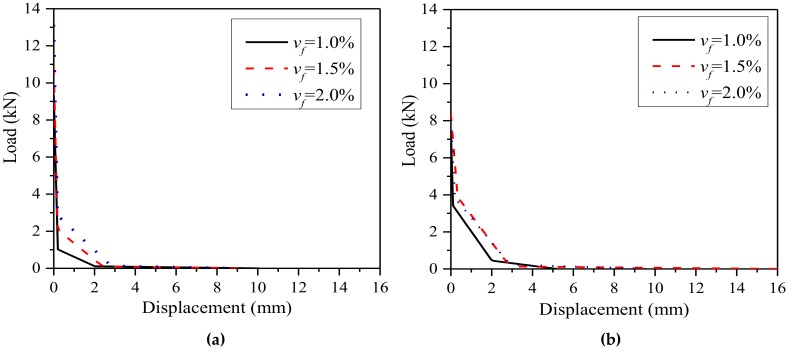
Trilinear strain-softening diagrams of steel fiber-reinforced SGM/SGC. (**a**) SGM; (**b**) SGC.

Figure 13 shows the trilinear strain-softening curves of steel fiber-reinforced SGM and SGC with three fiber volume contents obtained from the inverse analysis. It can be seen from Figure 13a that the second descending slope of the softening curve of steel fiber-reinforced SGM becomes higher with the increase of the fiber volume content. However, for the steel fiber-reinforced SGC, as shown in Figure 13b, this tendency is broken at the fiber volume content of 1.5%, which is consistent with the experimental results showing that 1.5% fiber content exhibits the best reinforcement.

**Figure 14:**
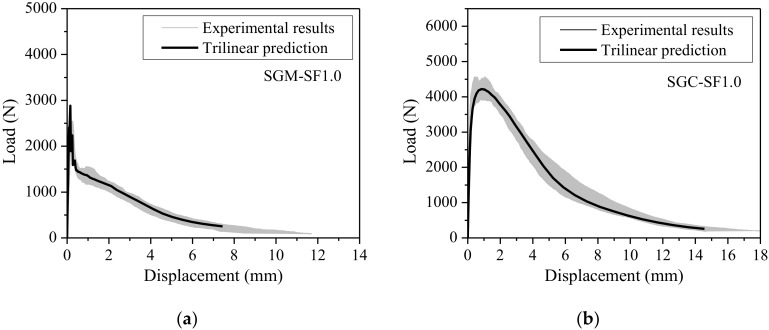
*P-δ* curves of fiber-reinforced SGM/SGC obtained from experiment and prediction. (**a**) SGM; (**b**) SGC.

The load-displacement curves of steel fiber-reinforced SGM and SGC with fiber volume content of 1.0% predicted by using the obtained trilinear strain-softening diagrams and directly obtained from experiments are shown in Figure 14. The shadowed areas represent the scatter of the experimental load-displacement curves of four identical specimens. It is evident from Figure 14 that the predicted load-displacement curves agree well with the experimental results, demonstrating the credibility of the trilinear softening curves obtained from the inverse analysis. 

## 4. Conclusions

The mechanical and fracture characteristics of plain and hooked-end steel fiber-reinforced slag-based geopolymer mortar and concrete (SGM and SGC) are analyzed and compared in this paper, and the following conclusions can be drawn.

(1) The inclusion of steel fibers increases both the compressive strength and the splitting tensile strength of SGM and SGC, while the reinforcing efficiency is more significant on the splitting tensile strength. The enhancements of the compressive and splitting tensile strengths increase with the fiber volume contents for SGM, while SGC has an optimal fiber volume content of 1.5%. In addition, the existing formula for steel fiber-reinforced PCC underestimates the splitting tensile strengths of steel fiber-reinforced SGC and SGM.
(2) The equivalent flexural strength *f_eq_*_,2_ of both SGM and SGC is higher than their other equivalent flexural strength *f_eq_*_,3_, and a linear trend between them is found. The residual flexural strength *f_R_*_,4_ is about 79% of the other residual flexural strength *f_R_*_,1_. A strong correlation between the equivalent and residual flexural strengths is also observed.
(3) The addition of steel fiber significantly improves the fracture energy of SGM and SGC. For SGM, the enhancement of fracture energy increases with the fiber volume content. The fracture energy of SGC reaches the largest value when the fiber volume content is 1.5%. The fracture toughness increases significantly with the fiber incorporation and the improvement can be more than four times for SGM with the fiber volume dosage of 2.0%.
(4) The trilinear strain-softening diagram can be used to predict the load-displacement curves of steel fiber-reinforced SGM and SGC.

## Figures and Tables

**Table 1:** Chemical composition of GGBFS.

**Chemical Content** **(% by weight)**	**CaO**	**Al_2_O_3_**	**S_i_O_2_**	**SO_3_**	**P_2_O_5_**	**MgO**	**Na_2_O**	**K_2_O**	**TiO_2_**
	33.3	16.9	33.4	2.35	3.77	7.0	2.0	0.16	0.61

**Table 2:** Properties and configuration of deformed steel fiber.

**Fiber Properties**	**Length (mm)**	**Diameter (μm)**	**Aspect Ratio**	**Tensile Strength (MPa)**	**Elastic Modulus (GPa)**	**Fiber Configuration** 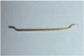
	13	212	60	≥2850	210	

**Table 3:** Mix proportions of SGM and SGC.

Mix Type	*n* (%)	*M_s_*	Slag kg/m^3^	Fine Aggregate kg/m^3^	Coarse Aggregate kg/m^3^	Water kg/m^3^	Alkali Activator		w/b	Sand Ratio
							Sodium Silicate Solution kg/m^3^	Sodium Hydroxide kg/m^3^		
SGC	4.5	2.0	420	694	1041	117	117	11	0.45	0.40
SGM	5.0	1.5	783	1174	-	254	182	30	0.44	-

Note: “*n*” is the alkali concentration and “*M_s_*” is the modulus of alkali activator.

**Table 4:** Average compressive and splitting tensile strengths of unreinforced and steel fiber-reinforced SGM and SGC.

Mortar/Concrete	*V_f_* (%)	*f_c_* (MPa)	Relative *f_c_* (%)	*f_st_* (MPa)	Relative *f_st_* (%)
SGM	-	69.2 ± 3.7	100	5.03 ± 1.7	100
SGM-SF1.0	1.0	78.0 ± 2.3	113	9.34 ± 1.1	186
SGM-SF1.5	1.5	78.2 ± 4.5	113	10.8 ± 0.7	215
SGM-SF2.0	2.0	84.6 ± 3.5	122	13.1 ± 1.3	261
SGC	-	69.9 ± 2.1	100	6.06 ± 0.9	100
SGC-SF1.0	1.0	81.1 ± 5.6	116	7.47 ± 1.6	123
SGC-SF1.5	1.5	85.6 ± 4.9	122	8.37 ± 0.6	138
SGC-SF2.0	2.0	78.9 ± 4.2	113	8.06 ± 1.4	133

**Table 5:** Fracture energy of plain and steel fiber-reinforced SGM/SGC.

Fracture Energy, *G*_F_ (N/m)	**SGM**	**SGM-SF1.0**	**SGM-SF1.5**	**SGM-SF2.0**
	97.4	994.9	2472.0	4750.8
	91.2	1368.2	2452.6	3542.2
	101.9	1341.3	2938.9	4585.3
	77.1	1148.1	2518.6	3873.6
Average *G_F_* (N/m)	91.9	1213.1	2595.5	4188.0
K*	1	13.2	28.2	45.9
Fracture Energy, G_F_ (N/m)	**SGC**	**SGC-SF1.0**	**SGC-SF1.5**	**SGC-SF2.0**
	207.4	4884.5	6626.3	6203.8
	203.0	3717.3	4874.0	5037.4
	213.6	4102.0	5626.6	5048.0
	207.4	3996.8	6373.9	5731.3
Average *G_F_* (N/m)	207.9	4175.1	5875.2	5505. 1
K*	1	20.1	28.2	26.5

*G_F_ (fiber-reinforced)/*G_F_* (non-reinforced)

**Table 6:** TPB tests: flexural strength parameters.

Content	*f_f,L_* (MPa)	*f_eq_*_,2_ (MPa)	*f_eq_*_,3_ (MPa)	*f_R_*_,1_ (MPa)	*f_R_*_,4_ (MPa)
SGC-SF1.0	4.69	7.81	6.71	6.88	5.00
SGC-SF1.5	5.55	9.22	8.76	8.35	7.13
SGC-SF2.0	4.66	8.18	7.86	7.51	6.64
SGM-SF1.0	1.23	3.27	2.29	2.50	1.49
SGM-SF1.5	2.55	5.50	4.44	4.79	3.04
SGM-SF2.0	4.23	6.76	6.07	6.29	4.83

**Table 7:** Fracture toughness and reinforcing efficiency of steel fiber-reinforced SGM and SGC.

Mortar	*K*_IC_ (MPa·m^1/2^)	*F*_T_	Concrete	*K*_IC_ (MPa·m^1/2^)	*F*_T_
SGM	1.01	1.00	SGC	1.71	1.00
SGM-SF1.0	1.35	1.34	SGC-SF1.0	4.79	2.80
SGM-SF1.5	2.03	2.01	SGC-SF1.5	5.92	3.46
SGM-SF2.0	4.29	4.25	SGC-SF2.0	5.24	3.06

**Table 8:** Trilinear softening curve parameters of steel fiber-reinforced SGM/SGC.

Mortar/Concrete	*f*_t_	*ω*_0_	*f*_1_	*ω*_1_	*f*_2_	*ω*_2_
SGC-SF1.0%	7.47	5.20	3.57	0.008	0.448	2.01
SGC-SF1.5%	8.37	16.47	4.36	0.006	0.134	2.88
SGC-SF2.0%	8.06	9.95	4.09	0.001	0.231	2.90
SGM-SF1.0%	9.34	10.05	1.12	0.008	0.109	2.01
SGM-SF1.5%	10.82	9.36	2.25	0.006	0.106	2.37
SGM-SF2.0%	13.14	9.08	3.04	0.001	0.128	2.82

## References

[1] Ding Y., Yu J.T., Yu K.Q., Xu S.L. (2018). Basic mechanical properties of ultra-high ductility cementitious composites: From 40 MPa to 120 MPa. Compos. Struct..

[2] Ding Y., Yu K.Q., Yu J.T., Xu S.L. (2018). Structural behaviors of ultra-high performance engineered cementitious composites (UHP-ECC) beams subjected to bending-experimental study. Constr. Build. Mater..

[3] Yu K., Li L., Yu J., Xiao J., Ye J., Wang Y. (2018). Feasibility of using ultra-high ductility cementitious composites for concrete structures without steel rebar. Eng. Struct..

[4] Wang S.D., Scrivener K.L., Pratt P.L. (2014). Factors affecting the strength of alkali-activated slag. Cem. Concr. Res..

[5] Palomo A., Grutzeck M.W., Blanco M.T. (1999). Alkali-activated fly ashes: A cement for the future. Cem. Concr. Res..

[6] Roy D.M. (1999). Alkali-activated cements opportunities and challenges. Cem. Concr. Res..

[7] Hardjito D., Wallah S.E., Sumajouw D.M., Rangan B.V. (2004). On the development of fly ash-based geopolymer concrete. ACI Mater. J..

[8] Purdon A.O. (1940). The action of alkalis on blast-furnace slag. J. Soc. Chem. Ind..

[9] Shi C., Roy D., Krivenko P. (2006). Alkali-Activated Cements and Concretes.

[10] Ding Y., Dai J.G., Shi C.J. (2016). Mechanical properties of alkali-activated concrete: A state-of-the-art review. Constr. Build. Mater..

[11] Wang S.D., Pu X.C., Scrivener K.L., Pratt P.L. (1995). Alkali-activated slag cement and concrete: A review of properties and problems. Adv. Cem. Res..

[12] Collins F.G., Sanjayan J.G. (1999). Workability and mechanical properties of alkali activated slag concrete. Cem. Concr. Res..

[13] Collins F.G., Sanjayan J.G. (2000). Cracking tendency of alkali-activated slag concrete subjected to restrained shrinkage. Cem. Concr. Res..

[14] Collins F.G., Sanjayan J.G. (2001). Microcracking and strength development of alkali activated slag concrete. Cem. Concr. Compos..

[15] Atiş C.D., Bilim C., Çelik O., Karahan O. (2009). Influence of activator on the strength and drying shrinkage of alkali-activated slag mortar. Constr. Build. Mater..

[16] Bernal S., Mejía de Gutierrez R., Rodriguez E., Delvasto S., Puertas F. (2009). Mechanical behavior of steel fibre-reinforced alkali activated slag concrete. Mater. Constr..

[17] Bernal S., De Gutierrez R., Delvasto S., Rodriguez E. (2010). Performance of an alkali-activated slag concrete reinforced with steel fibers. Constr. Build. Mater..

[18] Aydın S., Baradan B. (2013). The effect of fiber properties on high performance alkali-activated slag/silica fume mortars. Compos. Part B Eng..

[19] Puertas F., Gil-Maroto A., Palacios M., Amat T. (2006). Alkali-activated slag mortars reinforced with AR glassfibre. Performance and properties. Mater. Constr..

[20] Alcaide J.S., Alcocel E.G., Puertas F., Lapuente R., Garcés P. (2007). Carbon fibre-reinforced, alkali-activated slag mortars. Mater. Constr..

[21] Zegardło B., Szeląg M., Ogrodnik P. (2016). Ultra-high strength concrete made with recycled aggregate from sanitary ceramic wastes-The method of production and the interfacial transition zone. Constr. Build. Mater..

[22] Del Bosque I.S., Zhu W., Howind T., Matías A., de Rojas M.S., Medina C. (2017). Properties of interfacial transition zones (ITZs) in concrete containing recycled mixed aggregate. Cem. Concr. Compos..

[23] Siddique S., Shrivastava S., Chaudhary S. (2017). Lateral force microscopic examination of interfacial transition zone in ceramic concrete. Constr. Build. Mater..

[24] Wang X.H., Jacobsen S., He J.Y., Zhang Z.L., Lee S.F., Lein H.L. (2009). Application of nanoindentation testing to study of the interfacial transition zone in steel fiber reinforced mortar. Cem. Concr. Res..

[25] Shi C., Xie P. (1998). Interface between cement paste and quartz sand in alkali-activated slag mortars. Cem. Concr. Res..

[26] San Nicolas R., Provis J.L. (2012). Interfacial transition zone in alkali-activated slag concrete. Proceeding of the 12th International Conference on Recent Advances in Concrete Technology and Sustainability Issues.

[27] Castel A., Foster S.J. (2015). Bond strength between blended slag and Class F fly ash geopolymer concrete with steel reinforcement. Cem. Concr. Res..

[28] Nataraja M.C., Dhang N., Gupta A.P. (1999). Stress–strain curves for steel-fiber reinforced concrete under compression. Cem. Concr. Compos..

[29] Zollo R.F. (1997). Fiber-reinforced concrete: An overview after 30 years of development. Cem. Concr. Compos..

[30] Bhutta A., Borges P.H., Zanotti C., Farooq M., Banthia N. (2017). Flexural behavior of geopolymer composites reinforced with steel and polypropylene macro fibers. Cem. Concr. Compos..

[31] RILEM, FMC1 (1994). Determination of the Fracture Energy of Mortar and Concrete by Means of Three-Point Bend Tests on Notched Beams, RILEM Technical Recommendations for the Testing and Use of Construction Materials, E and FN SPON.

[32] Ding Y., Dai J.G., Shi C.J. (2018). Fracture properties of alkali-activated slag and ordinary Portland cement concrete and mortar. Constr. Build. Mater..

[33] Ding Y., Dai J.G., Shi C.J. (2018). Mechanical Properties of Alkali-Activated Concrete Subjected to Impact Load. J. Mater. Civ. Eng..

[34] Brough A.R., Atkinson A. (2002). Sodium silicate-based, alkali-activated slag mortars: Part I. Strength, hydration and microstructure. Cem. Concr. Res..

[35] (2003). Standard for Test Method of Mechanical Properties in Ordinary Concrete.

[36] Bharatkumar B.H., Raghuprasad B.K., Ramachandramurthy D.S., Narayanan R., Gopalakrishnan S. (2005). Effect of fly ash and slag on the fracture characteristics of high performance concrete. Mater. Struct..

[37] Rilem T.C. (2002). 162-TDF. Test and design methods for steel fibre reinforced concrete. Mater. Struct..

[38] Shah S.P. (1990). Determination of fracture parameters (K Ic s and CTOD c) of plain concrete using three-point bend tests. Mater. Struct..

[39] Sahmaran M., Yaman I.O. (2007). Hybrid fiber reinforced self-compacting concrete with a high-volume coarse fly ash. Constr. Build. Mater..

[40] Dawood E.T., Ramli M. (2011). High strength characteristics of cement mortar reinforced with hybrid fibres. Constr. Build. Mater..

[41] Song P.S., Hwang S. (2004). Mechanical properties of high-strength steel fiber-reinforced concrete. Constr. Build. Mater..

[42] Xu B.W., Shi H.S. (2009). Correlations among mechanical properties of steel fiber reinforced concrete. Constr. Build. Mater..

[43] Sarker P.K. (2011). Bond strength of reinforcing steel embedded in fly ash-based geopolymer concrete. Mater. Struct..

[44] Ozalp F., Akkaya Y., Sengul C., Akcay B., Tasdemir M.A., Kocaturk A.N. (2007). Curing Effects on Fracture of High Performance Cement Based Composites With Hybrid Steel Fibers. Proceedings of the 6th International Conference on Fracture Mechanics of Concrete and Concrete Structures.

[45] Lin T., Jia D., He P., Wang M., Liang D. (2008). Effects of fiber length on mechanical properties and fracture behavior of short carbon fiber reinforced geopolymer matrix composites. Mater. Sci. Eng. A.

[46] Barros J.A., Cunha V.M., Ribeiro A.F., Antunes J.A. (2005). Post-cracking behaviour of steel fibre reinforced concrete. Mater. Struct..

[47] Rosenbusch J., Teutsch M., Schnütgen B., Vandewalle L. (2003). Shear design with “σ-ε-Method”. International RILEM Workshop on Test and Design Methods for Steelfibre Reinforced Concrete.

[48] Tada H., Paris P.C., Irwin G.R. (2000). The Stress Analysis of Cracks Handbook.

[49] Dias D.P., Thaumaturgo C. (2005). Fracture toughness of geopolymeric concretes reinforced with basalt fibers. Cem. Concr. Compos..

[50] Nematollahi B., Sanjayan J., Shaikh F.U.A. (2014). Comparative deflection hardening behavior of short fiber reinforced geopolymer composites. Constr. Build. Mater..

[51] Kizilkanat A.B., Kabay N., Akyüncü V., Chowdhury S., Akça A.H. (2015). Mechanical properties and fracture behavior of basalt and glass fiber reinforced concrete: An experimental study. Constr. Build. Mater..

[52] Hillerborg A., Modéer M., Petersson P.E. (1976). Analysis of crack formation and crack growth in concrete by means of fracture mechanics and finite elements. Cem. Concr. Res..

[53] Kazemi M.T., Fazileh F., Ebrahiminezhad M.A. (2007). Cohesive crack model and fracture energy of steel-fiber-reinforced-concrete notched cylindrical specimens. J. Mater. Civ. Eng..

[54] Kang S.T., Lee Y., Park Y.D., Kim J.K. (2010). Tensile fracture properties of an Ultra High Performance Fiber Reinforced Concrete (UHPFRC) with steel fiber. Compos. Struct..

[55] Park K., Paulino G.H., Roesler J. (2010). Cohesive fracture model for functionally graded fiber reinforced concrete. Cem. Concr. Res..

[56] Roelfstra P.E., Wittmann F.H., Wittmann F.H. (1986). Numerical method to link strain softening with failure of concrete. Fracture Toughness and Fracture energy of Concrete.

[57] Yu K., Yu J., Lu Z. (2015). Determination of the softening curve and fracture toughness of high-strength concrete exposed to high temperature. Eng. Fract. Mech..

[58] Yu K.Q., Yu J., Lu Z. (2016). Fracture properties of high-strength/high-performance concrete (HSC/HPC) exposed to high temperature. Mater. Struct..

[59] Villmann B., Villmann T., Slowik V. (2004). Determination of softening curves by backward analyses of experiments and optimization using an evolutionary algorithm. Proceedings of the 5th International Conference on Fracture Mechanics of Concrete and Concrete Structures.

[60] Slowik V., Villmann B., Bretschneider N., Villmann T. (2006). Computational aspects of inverse analyses for determining softening curves of concrete. Comput. Methods Appl. Mech. Eng..

